# Mini-track, mini-nephroscopy, mini-ultrasonic probe percutaneous nephrolithotomy and its initial clinical application

**DOI:** 10.1186/s12894-022-01061-0

**Published:** 2022-09-07

**Authors:** Yang Hong, Huanrui Wang, Qingquan Xu, Liang Chen, Xiaobo Huang, Liulin Xiong

**Affiliations:** 1 The Department of Urology, Peking University People’s Hospital, 11# Xizhimen Nandajie Street, XiCheng District, Beijing, 100034 China; 2 Peking University Applied Lithotripsy Institute, Beijing, China

**Keywords:** Percutaneous nephrolithotomy, Ultrasonic probe, Kidney calculi, Stone free rate, Complication

## Abstract

**Background:**

To assess the outcome of the mini-track, mini-nephroscopy, mini ultrasonic probe percutaneous nephrolithotomy for upper ureteral and kidney stones.

**Methods:**

We collected data of 53 patients (55 kidney units) who underwent mini-track, mini-nephroscopy, mini-ultrasonic probe percutaneous nephrolithotomy between September 2020 and March 2021. The study included single and upper ureteral stones from 12 kidneys, multiple stones from 28 kidneys, and staghorn stones from 15 kidneys.

**Results:**

The mean operative duration was 50.6 min, ranging from 15 to 200 min, whereas the mean lithotripsy and stone removal time was 17.2 min (3–45 min). Moreover, the mean postoperative length of stay was 4.0 days (1–7 days). Besides, the stone-free rate (SFR) of discharge was 89.1% (49/55). The mean hemoglobin drop was 15.3 mg/dL, ranging 1–32 mg/dL. Out of the total cases, only 4 of them displayed minor complications. The outcomes of < 40 mm versus ≥ 40 mm calculi were compared by performing subgroup analysis. The results demonstrated a longer operation duration (65.2 vs. 40.2 min), higher complication rate (13.0% vs. 3.3%), and lower SFR in the ≥ 40 mm calculi subgroup.

**Conclusions:**

In summary, mini-track, mini-nephroscopy, mini-ultrasonic probe percutaneous nephrolithotomy is an effective and safe method to treat patients with upper ureteral and kidney calculi. This is especially significant for the stone size of 20–40 mm, demonstrating excellent SFR and a lower complication rate.

## Background

Percutaneous nephrolithotomy (PCNL) is the preferred method for managing large and complex stones providing excellent stone-free rate (SFR) [1, 2]. With the development of miniaturization equipment, the risk and complication of PCNL have been reduced significantly using the small tract size (≤ 20 Fr). Nowadays, mini-PCNL can achieve comparable SFR compared to conventional PCNL, posing lower risks and complications related to the tract size [3]. There are several miniaturized PCNL available, such as ultra-mini PCNL (UMP), super-mini PCNL (SMP), needle-perc, and mini-perc, which use pneumatic or laser energy as the dominant method [4–6]. However, there is a paucity of studies that use ultrasound probes as the main lithotripsy method.

In our previous study, we examined the efficiency and safety of the micro-ultrasonic probe (2.0 mm) combined with ultrasound-guided mini-PCNL in managing upper ureteral and renal stones, especially for stones with size < 2 cm [7]. However, this approach showed limitations for large and complex stones. It has been observed that the efficiency of lithotripsy increases with the size of the ultrasonic probe. However, a large ultrasound probe requires a larger working tract and an outer diameter of the nephroscopy, increasing the size of the tract, thereby increasing the risks and complications of the operation, contrary to our original intention. Thus, we thought that designing a miniaturized nephroscopy to hold a larger-size ultrasound probe without significantly expanding the size of the tract would solve the problem. In the current study, we developed a miniaturized nephroscopy with an outer diameter of 12/15 Fr and a working channel of 10.5 Fr that could hold a 2.8 mm ultrasound probe for stone fragments and retrieval. Although we need to establish a tract of 18 Fr, this equipment demonstrated efficient lithotripsy and stone removal capacity, which is significantly higher than a 2 mm ultrasound probe. The miniaturized nephroscopy reduces the pressure in the renal pelvis during the operation, besides effectively managing complex and bulk stones. This procedure has three characteristics, named mini track (18 Fr), mini-nephroscopy (12/15 Fr), and mini-ultrasonic probe (2.8 mm). We conduct a study to assess the outcome of the mini-track, mini-nephroscopy, mini ultrasonic probe percutaneous nephrolithotomy for upper ureteral and kidney stones.

## Methods

Between September 2020 and March 2021, data on 53 consecutive patients with upper ureteral and renal stones treated with mini-track, mini-nephroscopy, mini-ultrasonic probe PCNL in a single tertiary institution by an experienced surgeon group were reviewed retrospectively. The criteria for using mini-track, mini-nephroscopy, mini-ultrasonic probe PCNL were based on (1) patients with stones resistant to ESWL or retrograde intrarenal surgery (RIRS) treatment, (2) stone size ≥ 2.0 cm or anatomic abnormality of the collecting system. The exclusion criteria: meet the inclusion criteria, but performed procedures other than mini-track, mini-nephroscopy, mini-ultrasonic probe PCNL.

All patients underwent detailed preoperative evaluation, including blood tests, urine analysis, stone characteristics (size, location, and composition), surgical details, and outcome. The characteristics of stones were evaluated using abdominal ultrasound (US), kidney, ureter, and bladder (KUB) X-ray, and non-contrast computed tomography (NCCT). Patients with positive preoperative urine cultures were treated with appropriate antibiotics, ensuring sterile urine during the procedure (Table 1). All patients with negative urine cultures were treated with single-dose broad-spectrum antibiotic prophylaxis before the mini-track, mini-nephroscopy, mini-ultrasonic probe PCNL. The mean stone size was evaluated using preoperative radiographs and determined by the largest diameter for a single stone. The summation of the diameter of the stones was used for kidneys with multiple stones.

**Table 1:** Demographics and stone characteristics of the patients that underwent Mini track, Mini-nephroscope, Mini ultrasonic probe percutaneous nephrolithotomy

Parameters	No.(%)	Mean ± SD (range)
Patients	53	
Kidney units	55	
Kidney left/right	35(63.7)/20(36.3)	
Gender male/female	40(75.5)/13(24.5)	
Age(year)		52.7 ± 12.1(23–78)
BMI(kg/m^2^)		26.4 ± 4.6(19.1–41.9)
Stone type		
Single	12(21.8)	
Multiple	28(50.9)	
Staghorn	15(27.3)	
No. stone location (%)		
Upper ureteral	6(10.9)	
Pelvis	4(7.3)	
Upper pole	2(3.6)	
Middle pole	2(3.6)	
Lower pole	4(7.3)	
Multiple locations	37(67.3)	
Stone size (cm)		
Preoperative urinary tract infections	14(26.4)	3.6 ± 1.4(1.2–6.7)
Kidney intervention history		
ESWL	13(23.6)	
RIRS	8(14.5)	
PCNL	11(20.0)	
Open surgery	1(1.8)	
Hydronephrosis(side)	36(65.5)	

### Armamentarium

The straight tube was contiguous with the access sheath and had a receptacle for a silicone or rubber cap at the proximal end. The left and right water inlet and outlet channel switch design made it more convenient for the surgery. The perfusion liquid via an irrigated side-port inflow passed through the internal surface of the 12/15 Fr oval sheath (9-F space). The irrigation port could be connected to an irrigation pump. Besides, the working channel could hold a 2.8 mm micro-ultrasound probe, pneumatic lithotripter probe, basket, or forceps.

### Procedure

**Fig. 1:**
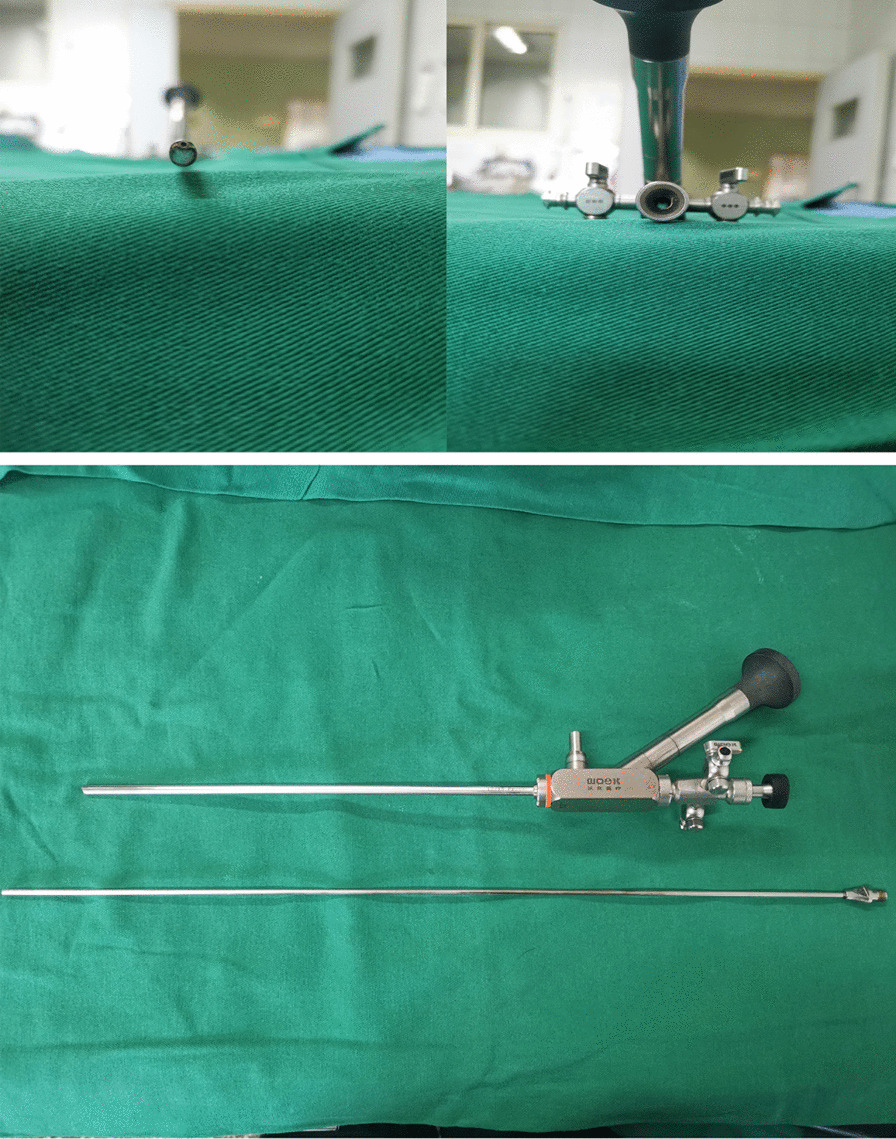
The equipment of 12/15Fr nephroscope and 2.8 mm ultrasonic probe

All patients underwent percutaneous puncture under spinal anesthesia (lumbar anesthesia or combined with spinal anesthesia). A retrograde 5F ureteral catheter (BARD, Inc. US) was inserted into the target kidney with a 22F cystoscope followed by a Foley catheter in the lithotomy position. The prone patients were punctured using a 17.5-gauge puncture needle following the US guidance. Next, a J-shaped guidewire was placed into the collecting system through the needle after observing the urine reflux. Nephrostomy tract dilatation was completed by a matched peel-away sheath (Cook, Inc. US) of 18 Fr through the guidewire. A 12/15 Fr rigid nephroscopy (WOOK Co., Ltd. China) with ultrasonic lithotripsy was used for stone fragmentation and removal (Fig. 1). Pneumatic lithotripter probe (Huifukang Co., Ltd. China) was only used for stones resistant to ultrasonic lithotripsy.

The energy and the duty ratio of ultrasonic lithotripsy (Huifukang Co., Ltd.) were set to 70%. Moreover, we set the flushing fluid flow rate to 500 mL/min and the vacuum suction pressure to 0.04–0.08 MPa.

The operation was said to be complete when the endoscopic and ultrasonic imaging detected no residual fragments. Nephrostomy tubes are usually placed in patients with significant bleeding or residual stone or fragments, which require a second-stage surgery. We placed a Double-J stent in patients as usual. Patients without nephrostomy tube and Double-J stent were defined as complete tubeless, whereas those with the Double-J stent left were defined as incomplete tubeless.

The operative time was calculated from the beginning of the renal puncture to wound suture or the nephrostomy tube placement. Postoperative blood tests were performed immediately. Hemoglobin loss was assessed at 24 h postoperatively. The KUB and/or NCCT scans were completed on day 1 post-operation, to observe the position of the Double-J stent and residual stones. Patients were considered stone-free if no stone or residual stone < 4 mm was present in the collective system or upper ureter. The SFR was calculated at hospital discharge and assessed using KUB and/or CT. The stone compositions were analyzed using infrared spectroscopy.

Patients with no nephrostomy tubes were still observed for renal perirenal effusion using the US after surgery. Furthermore, the nephrostomy tube was removed when no fever and pain occurred within 6 h, and the Foley catheter was removed the next day. All the Foley catheters and nephrostomy tubes were removed before patients were discharged. Complications were evaluated according to the Clavien-Dindo classification system [8]. Most Double-J stents were removed 2–4 weeks after the procedure.

### Statistical analysis

Data are reported as numbers, percentages, and mean ± standard deviation (SD). Differences between the groups were compared using the independent-samples t-test and chi-square test.

## Results

All patients underwent the mini-track, mini-nephroscopy, mini-ultrasonic probe PCNL under US guidance (Table 1). The stone was disintegrated into 1.0 mm fragments. The central region of the pole (47 renal units, 70.1%) was most commonly accessed (Table 2). Ten renal units (14.9%) in each upper and the lower pole were accessed (Table 2).

**Table 2:** The outcome of ultrasound-guided Mini track, Mini-nephroscope, Mini ultrasonic probe percutaneous nephrolithotomy

Parameters	No.(%)	Mean ± SD(range)
No. tract		
Single	46(83.6)	
Multiple	9(16.4)	
Puncture site		
Upper calyx	10(14.9)	
Middle calyx	47(70.2)	
Lower calyx	10(14.9)	
Operative time(min)		50.6 ± 37.6(15–200)
Postoperative hemoglobin drop(mg/dL)		15.3 ± 7.3(1–32)
Postoperative hospital time(d)		4.0 ± 1.8(1–7)
SFR at discharge	49/55(89.1)	
SFR of single stone	12/12(100)	
SFR of multiple stone	26/28(92.9)	
SFR of staghorn stone	11/15(73.3)	
Complications		
Grade I + II	4(7.5)	
Fever(> 38℃)	4 (7.5)	
Partial tubeless rate	20/55(36.4)	
No. site of puncture(%)		
Subcostal	49	
Intercostal	18	

In all, 67 tracts were established in 55 renal units of 53 patients. A single procedure was performed in fifty-two cases (98.1%), whereas one patient (1.9%) was operated on in two sessions. The mean operative time was 50.6 min (15–200 min). The mean hemoglobin drop was found to be 15.3 mg/dL (1–32 mg/dL). The mean postoperative discharge time was 4.0 days (1–7 days), and the SFR at discharge was 89.1%. The incomplete tubeless rate was 36.4%, while the complete tubeless rate was 0. Post-operative complications occurred in four patients (7.5%) with minor complications (Grades I and II), including fever (> 38 °C) managed without antibiotics in three patients and requiring additional antibiotics in one case. No patients were administered blood transfusion in our study.

Subgroup analysis was performed to compare the outcomes in < 4.0 cm versus ≥ 4.0 cm calculi (Table 3). The results demonstrated a longer operation time (40.2 vs. 65.2 min, *p* = 0.013), higher complication rate (13.0% vs 3.3%, *p* = 0.185), and lower SFR (73.9% vs. 100%, *p* = 0.002) in the ≥ 4.0 cm calculi subgroup compared with the < 4.0 cm group.

**Table 3:** Outcome of calculi treated with Mini track, Mini-nephroscope, Mini ultrasonic probe percutaneous nephrolithotomy

	Stone diameters		*p* value
	< 4 cm	≥ 4 cm	
No. of patients	30	23	–
No. of units	32	23	–
Mean stone size	2.6	5.0	–
Mean hospital stay	3.6	4.5	0.066
Modified Clavien complications	1	3	0.185
SFR	100	73.9	0.002
Mean operative time(minutes)	40.2	65.2	0.013
Mean hemoglobin loss(mg/dL)	14.8	16.7	0.364
Tubeless	15	5	0.056

Stone analysis was performed in 49 patients using infrared spectrophotometry. Results revealed the presence of calcium oxalate stone in 29 cases, mixed composition stone in 10 cases, struvite stones in five cases, uric acid stone in four cases, and calcite stone in one case.

## Discussion

In recent decades, the treatment of urolithiasis has undergone several changes. The treatment of calculi is mainly to balance the relationship between stone removal and surgical complications. As a low-risk treatment, ESWL often requires multiple treatments and residual stones [9, 10]. Although RIRS reduces the risk of bleeding and surrounding organ damage, it has its limitations [11–13].

Although standard PCNL is very effective (90%) for the treatment of stones of size > 20 mm, it also leads to some severe complications, such as bleeding. Severe bleeding may require arterial embolization, which may impair kidney function [14]. Previous studies have shown that the size of the percutaneous access tract is closely related to bleeding complications [3]. Mini-PCNL was first applied to children with kidney stones by Jackman in 1998 with the aim to reduce complications [15]. However, nowadays, mini-PCNL is also widely used in procedures for adults.

Four methods are commonly used for lithotripsy in nephroscopy or ureteroscopy, such as electrohydraulic, US, pneumatic, and laser lithotripsy. Electrohydraulic lithotripsy has excellent efficacy in treating most of the stones. However, it is more likely to cause complications, such as urothelial injury, hemorrhage, and tissue perforation [7]. Laser lithotripsy is another effective method but is also prone to complications related to heat injury and spontaneous stone [16–18]. US lithotripsy with a nephroscopy or ureteroscopy can facilitate the removal of small stone fragments by suction through a hollow probe [7, 19–21].

In the past, the PCNL puncture was done under the guidance of radiation; however, the surgeons and patients are exposed to radiation. According to the ICRP 60 report, the annual radiation dose for radiation workers is 20 mSv [22, 23]. Previous studies have shown that the mean radiation dose received during PCNL surgery was 8.66 mSv [23]. Ultrasound-guided puncture and tract establishment are, however, considered safe and effective, avoiding radiations, especially for young patients [7, 13]. In recent years, numerous reports demonstrate the success rate and complication rate of PCNL under ultrasound guidance similar to PCNL under radiation [24].

The efficacy and safety of any stone removal procedure are assessed by three criteria: (1) SFR, (2) complication rate, and (3) auxiliary procedure rate. In our study, the SFR was found to be 89.1%. Subgroup analysis showed that the mini-track, mini-nephroscopy, mini-ultrasonic probe PCNL was more effective in managing smaller (< 4.0 cm) rather than larger (≥ 4.0 cm) kidney calculi (SFR 100% vs. 73.9%). The overall rate of complication was 7.5%. Nevertheless, it is optimal for managing stones < 4.0 cm, even for partial staghorn stones. One RCT study demonstrated that mini-PCNL with 18Fr tract achieves a noninferior SFR compared to standard PCNL for the treatment of 20–40 mm renal stones, but with the advantages of reduced blood loss, less postoperative pain, and shorter hospitalization. Additionally, mini-PCNL does not cause an increase in the infectious complications [25].Some strategies for this procedure have been described before [7], such as the maintenance of coaxial operation and ultrasonic lithotripsy energy and duty ratio to 70%, combined with pneumatic for hard stone to avoid overload. The advantages include reduced difficulty and risk of the procedure, higher efficiency of lithotripsy and stone removal, reduced intrapelvic pressure during operation, relatively safe removal of calculus in the calyx with a narrow neck, and convenient operation. However, they are also associated with limitations, such as the speed of stone fragmentation being slightly lower than that of standard PCNL, and the water outlet may be affected when there is more bleeding during the operation.

However, the limitations of this study are in the retrospective data and the small cohort. Larger cohorts of randomized controlled studies should be conducted to support our findings.

## Conclusions

To summarize, the mini-track, mini-nephroscopy, mini-ultrasonic probe PCNL with US guidance is an effective and safe procedure for percutaneous nephrolithotomy of upper ureteral and kidney stones. Moreover, it may serve as an alternative method to RIRS or PCNL in managing stones of size 20–40 mm; however, further randomized controlled studies are warranted.

## Data Availability

The raw datasets used and/or analyzed during the current study are available from the corresponding author on reasonable request.

## Abbreviations

PCNL: Percutaneous nephrolithotomy
ESWL: Extracorporeal shock wave lithotripsy
SFR: Stone free rate
US: Ultrasound
CT: Computed tomography
KUB: Kidney-ureter-bladder plain
RIRS: Retrograde intrarenal surgery
UMP: Ultra-mini percutaneous nephrolithotomy
SMP: Super-mini percutaneous nephrolithotomy
